# Influence of SiC Sludge on the Microstructure of Geopolymers

**DOI:** 10.3390/ma13092203

**Published:** 2020-05-11

**Authors:** Kae-Long Lin, Kang-Wei Lo, Ta-Wui Cheng, Wei-Ting Lin, Ya-Wen Lin

**Affiliations:** 1 Department of Environmental Engineering, National Ilan University, Ilan 260, Taiwan; kllin@niu.edu.tw; 2 Graduate Institute of Engineering Technology, National Taipei University of Technology, Taipei 106, Taiwan; 3 Institute of Mineral Resources Engineering, National Taipei University of Technology, Taipei 106, Taiwan; twcheng@ntut.edu.tw (T.-W.C.); urine1001@gmail.com (Y.-W.L.); 4 Department of Civil Engineering, National Ilan University, Ilan 260, Taiwan; wtlin@niu.edu.tw

**Keywords:** SiC sludge, alkaline-activator solutions, NASH gels, Na_2_SiO_3_ solution/ sodium hydroxide solution mass ratio

## Abstract

There are considerable resource reuse and environmental concerns regarding SiC sludge (SiCS) that results from cutting silicon ingots into wafers. In the current study, the effect of the Na_2_SiO_3_ solution/sodium hydroxide solution (NS/SS) mass ratio and SiCS amount on metakaolin geopolymers was found during geopolymerization system performance. The results indicate that while NS/SS ratio was relatively low, increasing the NaOH content resulted in a sufficient amount of OH^−^ in the system to increase the solubility and hinder polycondensation, as indicated by the bulk density and setting-time results; since the polycondensation was inhibited, the mechanical strength was reduced. This study demonstrated that a geopolymer can be formed from a substitution of 10% SiCS and with an NS/SS ratio of 1.6, and that this geopolymer is a feasible material.

## 1. Introduction

Silicon carbide (SiC) is widely employed in high-temperature application [1,2] on account of its high strength properties as well as oxidation resistance. SiC is used in cutting tools [3] and armor [4]. Among its many applications in various arenas, SiC has been employed widely in cutting tool uses, especially for cutting silicon ingots. A large amount of SiC sludge (SiCS) is produced in cutting silicon ingots, causing serious environmental pollution. The kerf-loss of silicon waste is greater than 200,000 tons per year, and this sludge contributes to environmental contamination. Therefore, the recycling of SiCS could reduce environmental pressure and improve resource reuse. Moreover, the chemical properties of SiCS are the same as that of SiO_2_ and Al_2_O_3_. Therefore, the application of SiCS in enhancing the properties of geopolymers was studied.

Inorganic polymers (e.g., geopolymers) are cementation materials with the potential to be an alternative to ordinary Portland cement [5]. Geopolymers are mostly formed by mixing silicate aluminum materials (e.g., industrial waste, such as fly ash and GGBS (Ground-granulated blast-furnace slag)) and alkaline solutions or alkaline metals [6]. Commonly used alkaline solutions are Na_2_SiO_3_ and NaOH. However, alkaline-activator solutions play important roles in the polymerization process, which is strongly dependent on the Si/Al ratio, M_2_O/H_2_O ratio (where M = potassium or sodium), and hydroxide is used [7,8,9,10].

Lahoti et al. (2018) reported the microstructure as well as thermal performance of metakaolin geopolymer dependent on molar ratio. The sample with molar ratio 1.75 was of higher strength in that study, because viscous sintering of mixtures with a higher molar ratio at temperatures higher than 600 °C enabled local microcracks [11]. Zhang et al. (2019) reported that when the amount of NaOH is less than 10%, there is an increased sodium hydroxide content as a result of elastic-type properties by the Si–O–Na bonds, which creates variations in aluminosilicate gel [12]. Hu et al. (2019) reported that when the Na_2_O addition increased to 5 wt.%, the nepheline stage was acknowledged, and had soluble properties in the presence of alkaline medium to enhance the dissolution Al_2_O_3_ and SiO_2_ [13]. Wang et al. (2020) investigated molar ratio 1.5, and found that AlO_4_ content is higher and porosity is lower. The degree of efflorescence decreases with an increase in AlO_4_ content [14]. Hamdi et al. (2019) reported that increasing the percentage of phosphogypsum to 8% resulted in enhanced mechanical properties. The highest strength of 36 MPa was attained for an NaOH concentration of 14 M in that study [15]. Based on the literature review, it appears that there is a shortage of knowledge regarding the effects of SiCS on the mechanical and structural properties of geopolymers. The objective of this paper is to investigate the effect of an alkaline-activator solution on a geopolymer reaction of SiC sludge-based geopolymers (SiCSMG).

The present study investigated a geopolymerization system where geopolymers with SiCS contents of 0–40 %were produced. The Na_2_SiO_3_ solution/sodium hydroxide solution (NS/SS) mass ratios employed were 0.8–1.6. The properties of geopolymer, such as bulk density, compressive strength, setting time, and microstructure, were found.

## 2. Materials and Methods 

### 2.1. Materials and Methods

The starting materials were metakaolin (MK) and SiCS. The MK was produced through the calcination of SNOBRITE kaolin at temperature of 650 °C for 3 h. The SiCS was acquired from a plant that manufactures LED substrates. An elementary analysis of SiCS showed a make-up of 51.35% Si, 6.90% C, and 0.42% Al; the MK included 24.21% Si and 22.77% Al (Table 1). 

SiCS as well as MK samples had size less than 0.074 mm. A sodium hydroxide solution is prepared with 10 M and mixed to a Na_2_SiO_3_ with an Ms = 3.1 (SiO_2_ = 28.1%, Na_2_O = 9.09%, and H_2_O = 62.8%). The NS/SS ratios were 0.8, 1.2, and 1.6. Then, the MK and SiCS (0%–40% by weight) is included in alkali activator. The SiC sludge metakaolin-based geopolymer (SiCSMG) specimens are synthesized through the mixture of solid powder with alkali in mixer at 100 rpm up to 5 min. After that, paste is emptied into a plastic mold (size = 25.4 × 25.4 × 25.4 mm^3^) for analysis of the microstructure characteristics and mechanical properties. Specimens are then cured at 30 °C. The workability of the SiCSMGs were analyzed based on the ASTM C191 standard [16]. The mechanical strength of the SiCS samples was analyzed after 1, 7, 14, 28, and 56 days based on the ASTM C39/C39M-20 standard [17]. The mechanical properties were evaluated by the three specimens and are reported herein. Finally, the bulk density, porosity, FTIR (Fourier Transform Infrared) spectroscopy results, and SEM (Scanning Electron Microscope) images of the SiCSMGs were obtained at appropriate curing times, as follows. The bulk density and porosity of the samples was analyzed after 1, 7, 14, 28, and 56 days. The FTIR spectroscopy of the samples was analyzed after 1, 7, 28, and 56 days. The SEM images of the samples was analyzed after 1, 7, and 56 days.

### 2.2. Analysis

X-Ray fluorescence (XRF) was performed with an automated RIX 2000 spectrometer (NEX CG ED-XRF, Rigaku, Tokyo, Japan) and verified the elementary analysis of the materials. The specimens were prepared for XRF analysis by mixing 0.4 g of the sample and 4 g of 100 Spectroflux, at a dilution ratio of 1:10. The bulk density and porosity of the geopolymers were tested according to the Archimedes principle. The samples were placed in a beaker of distilled water for 24 h to get saturated samples. The suspension weight (W_1_) was measured as the weight of samples suspended in water, then the samples were removed from the water and the surface drops were dried and the saturated weight (W_2_) was measured in air. Finally, the specimens were placed in an oven at 105 ± 5 °C and dried to constant weight, and then measured the dried weight (W_3_) after cooling. Three specimens were used for the bulk density and porosity tests while the average value from the three specimens is presented. Therefore, the bulk density and porosity of the geopolymers samples can be obtained by the following formulas. Volume of the specimen (V (cm^3^)) = W_2_−W_1_; Bulk density (g/cm^3^) = W_3_/V; Porosity (%) = (W_2_−W_3_)/V) × 100%. Fresh-paste setting time was determined using a Vicat apparatus (63-L0028/A, CONTROLS, Liscate, Italy) for the workability of the SiCSMGs. Compressive strength was performed using a SUMMIT KH-3000 (Summit Kh-3000, Cochin Enterprise, Taipei, Taiwan), which was tested using a 50 mm^3^ cube. Three cubes from each sample were tested. The average strength value of the three specimens is presented. The coefficient of variation of these results was less than 10%. FTIR spectrometry was performed using a Bomem DA8.3 instrument (PerkinElmer FTIR TWO, PerkinElmer, New York, NY, USA). FTIR spectra were obtained by scanning 2000 cm^−1^ to 400 cm^−1^ wavenumbers using the KBr pellet technique (where 1 mg powdered sample was mixed with 150 mg KBr). SEM images were obtained using a Hitachi S-3500N (Hitachi S-3500N, Hitachi, Barcelona, Spain) to show the geopolymer microstructure.

## 3. Results

### 3.1. Mechanical Characteristics of the SiCSMGs

**Figure 1:**
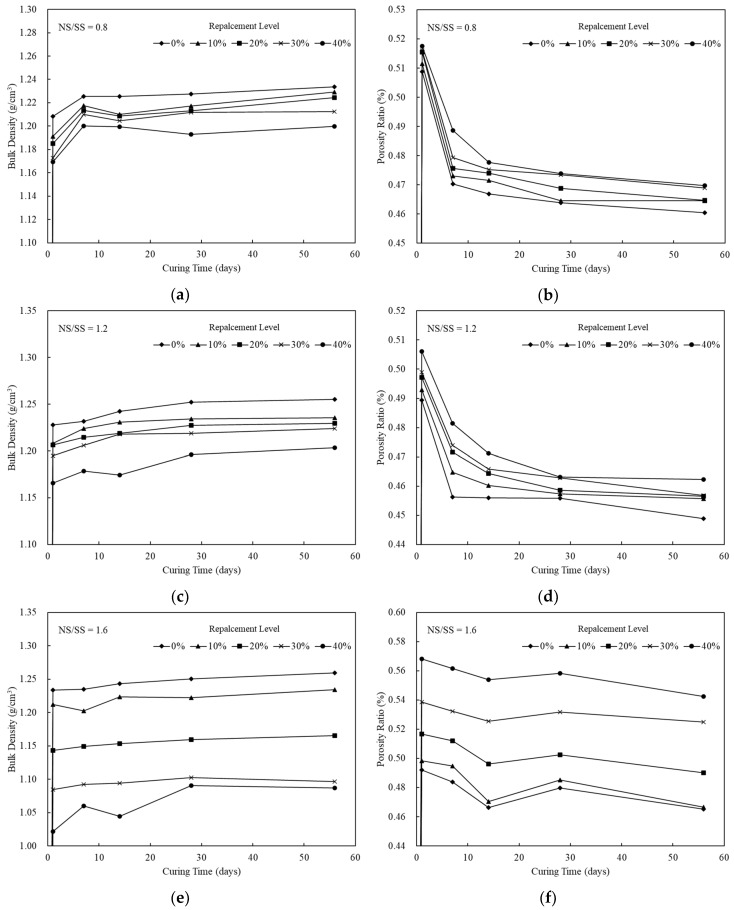
Bulk Density and Porosity of SiCSMGs for different NS/SS ratios: (**a**) Bulk Density for NS/SS = 0.8; (**b**) Porosity for NS/SS = 0.8; (**c**) Bulk Density for NS/SS = 1.2; (**d**) Porosity for NS/SS = 1.2; (**e**) Bulk Density for NS/SS = 1.6; (**f**) Porosity for NS/SS = 1.6.

Figure 1 illustrates the effects of SiCS content (0%–40%), NS/SS ratio (0.8–1.6), and curing time (1–56 days) on the bulk density of the SiCSMGs. For an NS/SS ratio of 0.8 and 28 days curing, samples were prepared with 0%, 10%, 20%, 30%, and 40% SiCS contents with bulk density are 1.23, 1.22, 1.21, 1.21, and 1.19 g/cm^3^, respectively (Figure 1a). This reveals that the bulk density decreased as the SiCS replacement level increased because for a geopolymer paste with an elevated SiCS content, the amount of precursor in the system is low, which delays geopolymerization. As the bulk density increases, the mechanical strength of geopolymer also increases as showed by Heah et al. (2012), because the Na_2_SiO_3_/NaOH ratio and highly soluble initial solid content increased [18]. At the early stage of curing (i.e., the first day) for the NS/SS ratio = 1.2, the bulk density of the SiCSMGs was 1.21 g/cm^3^ for a SiCS content from 10% to 20% (Figure 1c). A bulk density of the SiCSMGs with 10%–20% SiCS content was measured as 1.23 g/cm^3^ after 28 days. Thus, as the curing time as well as SiCS content increased, and the bulk density also increased. When the NS/SS ratio was 1.6, the bulk density of the SiCSMG with 10% SiCS was 1.20 g/cm^3^ after 7 days of curing, as shown in Figure 1e.

Figure 1 shows the porosity of sample prepared with 0%–40% SiCS and NS/SS ratios of 0.8–1.6. The geopolymers had an NS/SS ratio of 0.8, and the porosity of the geopolymers was 46.45%–46.98% for SiCS content of 10%–40%, suggesting that increasing the SiCS content affected the geopolymer porosity after 56 days. This finding was for a higher SiCS content in geopolymer paste, reducing the amount of precursor and causing delay in geopolymerization, leading to a decrease in the geopolymer product. The porosity of the 10% SiCSMGs with an NS/SS ratio of 1.6 was 46.67%; this may be due to geopolymer specimen pores filled.

### 3.2. Setting Time of the SiCSMGs

Table 2 details the setting time of the SiCSMGs with different NS/SS ratios. When the NS/SS ratio was 0.8 and the SiCS content was increased from 10% to 40%, the setting time (initial and final) also increased from 41 to 132 min and from 90 to 210 min, respectively (Table 2). The results indicate that at the periphery of a SiCS particle, surface geopolymer gel precipitate results in limited silica and alumina from metakaolin being dissolved, which causes a reduction to the precursor in the system, delaying the geopolymerization process, and an increasing to the setting time.

Table 2 shows that the NS/SS ratio increased by 1.2 to 1.6, resulting in extended workability. Therefore, in the SiCSMG with 10% SiCS replacement, the setting time (both initial and final) was also increased from 89 to 134 min and from 150 to 180 min, respectively (Table 2). Increasing the NS/SS ratio inhibited release of SiO_4_ and AlO_4_ from the SiCS and MK, reducing the number of silicon and aluminum ions released and forming Al(OH)_4_^−^as well as Si(OH)_4_^−^. Finally, replacing 40% of the SiCSMG with SiCS and using an NS/SS ratio of 1.6 resulted in increasing the initial to final set times from 145 to 221 min and from 210 to 285 min, respectively. Therefore, the high SiCS content of geopolymer sample may reduce the precursor amounts and delay the geopolymer reaction.

### 3.3. Compressive Strength Development of the SiCSMGs

**Figure 2:**
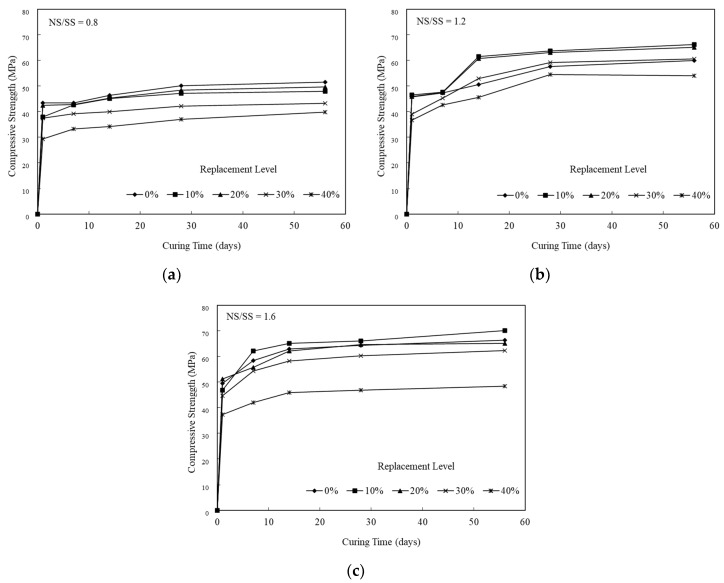
Comparison of the average compressive strength results for the SiCSMGs: (**a**) NS/SS = 0.8; (**b**) NS/SS = 1.2; (**c**) NS/SS = 1.6.

Figure 2 illustrates a compressive strength of the SiCSMGs with certain SiCS contents (0%–40%), NS/SS ratios (0.8–1.6), and curing times (1–56 days). At an NS/SS ratio of 0.8, the strength of the SiCSMG increased rapidly from 43.4 to 46.4 MPa in early curing time. The strength of the SiCSMG with 0% SiCS content was 51.5 MPa (Figure 2a after 56 days). When the NS/SS ratio was 0.8, the SiCSMG was weaker than those with higher NS/SS ratios (1.2–1.6) because increasing the NaOH content resulted in a sufficient amount of OH^−^ in the system to increase the solubility, which hindered the polycondensation reaction [19]; since polycondensation was inhibited, mechanical strength is reduced.

At an NS/SS ratio of 1.6, the SiCSMG with 10% SiCS had a higher strength of 70 MPa after 56 days. Increasing the NS/SS ratio promoted the dissolution of the SiCS and MK, accelerating the geopolymer reaction and prompting the constitution of networked geopolymer structures, as shown in Figure 2c. Therefore, the strength increased when additional amorphous geopolymer gel was formed in the geopolymerization system. Panias et al. (2007) noted that the geopolymer strength was connected to polycondensation, which affected SiO_4_ and AlO_4_ soluble content. A high degree of polycondensation in the Si–O–Al framework structure corresponded to a high strength [20,21].

### 3.4. FTIR Spectroscopy of the SiCSMGs

**Figure 3:**
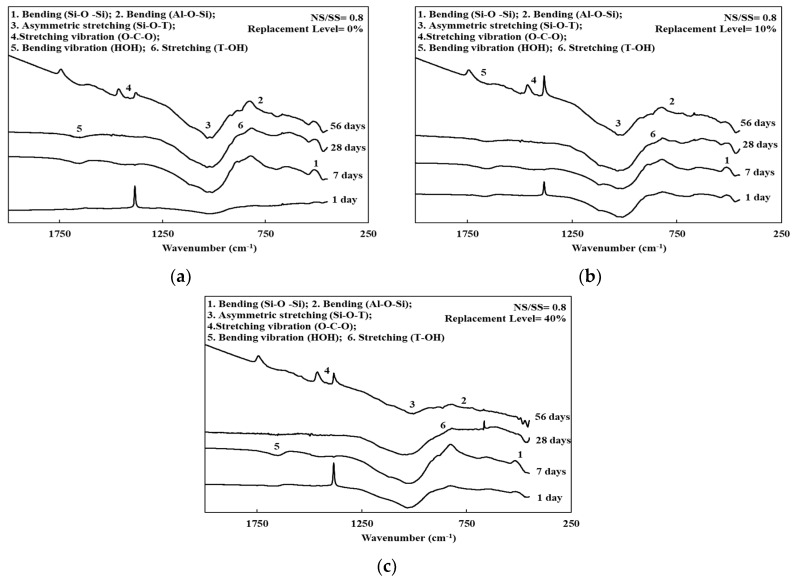
FTIR spectra of the SiCSMGs (NS/SS = 0.8): (**a**) Replacement Level = 0%; (**b**) Replacement Level = 10%; (**c**) Replacement Level = 40%.

**Figure 4:**
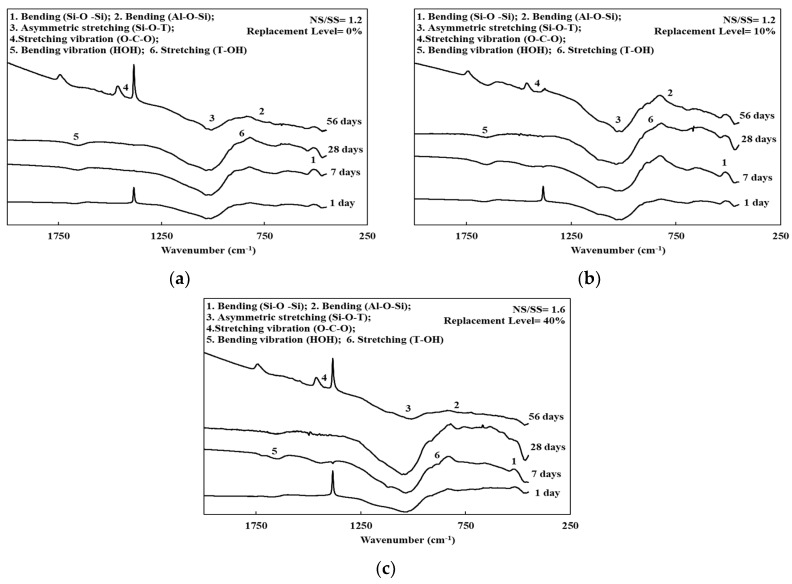
FTIR spectra of the SiCSMGs (NS/SS = 1.2): (**a**) Replacement Level = 0%; (**b**) Replacement Level = 10%; (**c**) Replacement Level = 40%.

**Figure 5:**
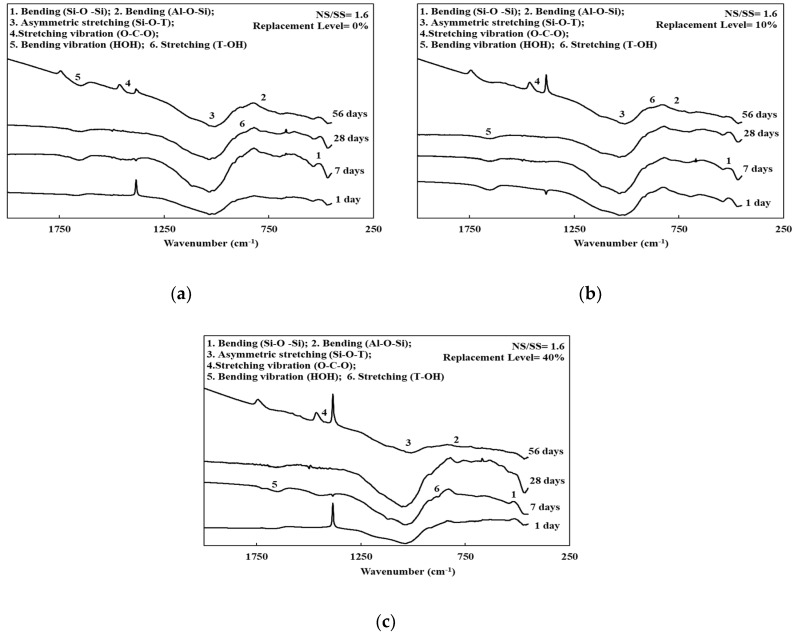
FTIR spectra of the SiCSMGs (NS/SS = 1.6): (**a**) Replacement Level = 0%; (**b**) Replacement Level = 10%; (**c**) Replacement Level = 40%.

Figure 3, Figure 4 and Figure 5 and Table 3 present the FTIR spectra of the SiCSMGs with various NS/SS ratios. It can be seen from Table 3 and Figure 3 that the SiCSMG spectrum obtained by curing till 1 day with a SiCS replacement is 0% and NS/SS ratio of 0.8 had some bands at 470, 700 and 1037 cm^−1^, corresponding to Si–O–Si bonds, Al–O–Si and Si–O–T (where T = Al or Si). The extending vibration of Si–O–T bond from 1037 to 1028 cm^−1^ with the curing time increasing to 56 days, which was observed in geopolymerization.

Figure 4 and Figure 5 show the higher a NS/SS ratio of 1.2–1.6 in the SiCSMGs with a SiCS replacement ratio of 10% resulting in a shift of the O–H stretching vibration band from 1655 to 1646 cm^−1^, corresponding to H_2_O bonding in the N–A–S–H (sodium aluminosilicate hydrate) gels [22]. With the high NS/SS ratio, the peak from the Si–O–T bond (asymmetric type) stretching at 1037 cm^−1^ transfer to 1027 cm^−1^ was a result of the accelerated dissolution and participation of unreacted particles during geopolymerization after up to 28 days of curing [23].

### 3.5. SEM Images of the SiCSMGs

**Figure 6:**
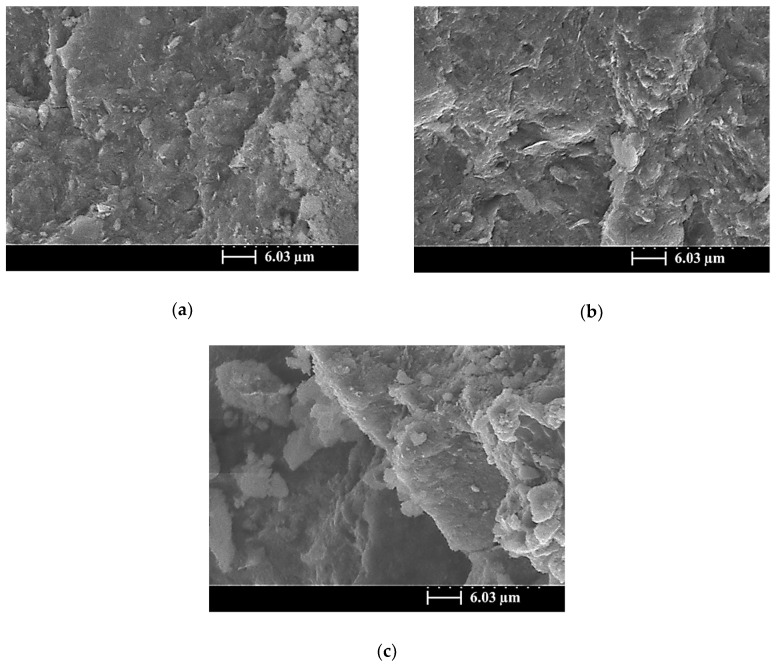
SEM image of a SiCSMGs after 1 day of curing (NS/SS = 1.6): (**a**) Replacement Level = 0%; (**b**) Replacement Level = 10%; (**c**) Replacement Level = 40%.

**Figure 7:**
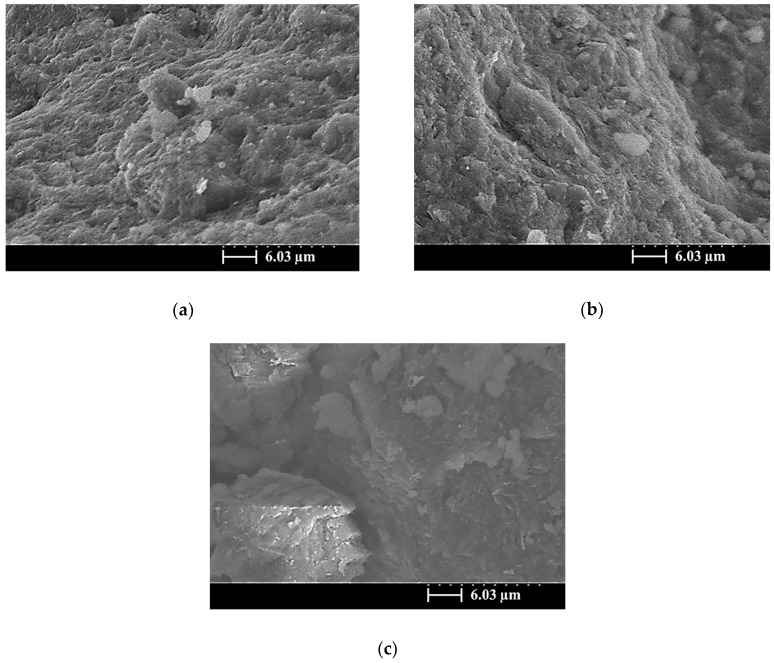
SEM image of a SiCSMGs after 7 days of curing (NS/SS = 1.6): (**a**) Replacement Level = 0%; (**b**) Replacement Level = 10%; (**c**) Replacement Level = 40%.

**Figure 8:**
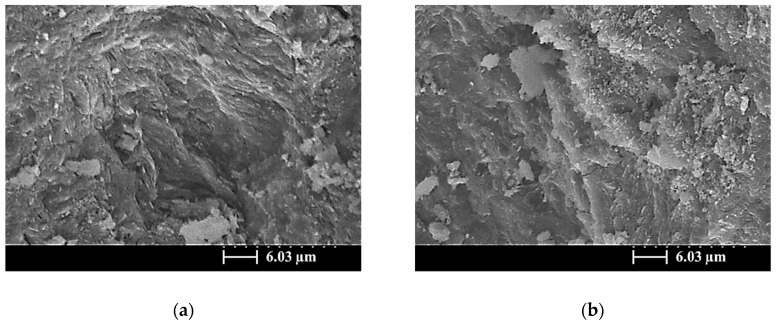

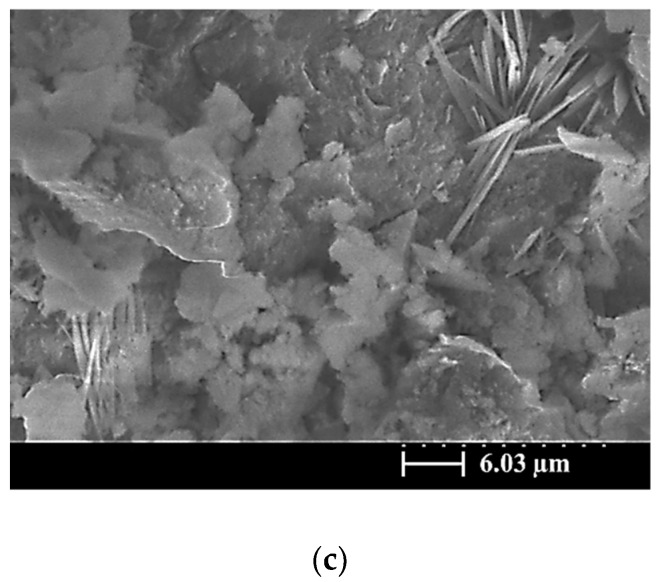
SEM image of a SiCSMGs after 56 days of curing (NS/SS = 1.6).: (**a**) Replacement Level = 0%; (**b**) Replacement Level = 10%; (**c**) Replacement Level = 40%.

Figure 6, Figure 7 and Figure 8 present SEM images of the SiCSMGs with an NS/SS ratio of 1.6 and various SiCS contents (0%–40%) after 1, 7, and 56 days of curing. In the SEM images, the SiCSMG with a SiCS replacement ratio of 0%–10% had a layered structure and angular edges, cured for 1 days (Figure 6a,b). Kong et al. (2007) suggested that geopolymer structures typically have angular edges, and this was confirmed by Sanjayan and Sageo-Crentsil [24]. Unreacted and flaked MK and SiCS particles are clearly visible in the SEM images of the sample with a SiCS replacement level of 40%; this behavior is believed to be a periphery of the SiCS particle surface geopolymer gel precipitate, resulting in limited silica and alumina from metakaolin being dissolved (Figure 6c). During the 7 days of curing, the SiCSMGs exhibited continuous geopolymer reaction and generation, and the amorphous gel products gradually filled the mesoporous areas of the geopolymer structure, resulting in a high density and improving the mechanical properties of the SiCSMGs.

At 56 days, the amorphous gel products had gradually filled the pores of the SiCSMGs, resulting in increase in density and compressive strength of the geopolymer structure, as shown in Figure 8. Panias et al. (2007) noted that the strength property is connected to polycondensation. A high degree of polycondensation in the Si–O–Al framework structure corresponds to high strength [20]. These results were typical of physical properties and FTIR analysis; thus, the increase in density was also observed in the geopolymer with 40% SiCS (Figure 8c at 56 days). This is consistent with the results of previous studies that have stated that the maximum compressive strength and lowest porosity for SiCSMGs occurs at a SiCS replacement level of 10%.

## 4. Discussion

Based on the objectives of this paper, relationships were obtained between the different parameters in the present study and comparison with other studies is as follows. As shown in Figure 2, the compressive strength of geopolymer samples at the NS/SS ratio of 0.8 was weaker than those at different NS/SS ratios (1.2–1.6). Because the NS/SS ratio of 0.8 had more NaOH content, this resulted in an excessive amount of OH^−^ in the system, which caused precipitations of geopolymer gels around the surface of SiCS and MK particles, and hindered the polycondensation reaction [19]; since the polycondensation was inhibited, the mechanical strength is reduced. Lo et al. [25] reported the effect of thin film transistor liquid crystal display waste glass (TLWG) (0–40 wt.%) and SiO_2_/Na_2_O (S/N) ratio on the properties of metakaolin-based geopolymers (MKGP) [24]. With increasing Na_2_O concentration in the activator solution, the solubility of silica and alumina in TLWG increased, but the amount of silica and alumina dissolved in TLWG increased is limited, which is due to precipitations of geopolymer gels around the surface of waste glass particles [25]. From the results, it is understood that when NS/SS ratio is relatively low, and when OH^−^ increased, the solubility is increased, which leads to an acceleration in the condensation as shown from the setting time (Table 2), but polycondensation is inhibited, which leads to reductions in mechanical properties, e.g., bulk density, porosity, and compressive strength (Figure 1 and Figure 2), which is similar to other studies [25].

On the other hand, the dissolved Si and Al ions content in the system was high, which may lead to the densification of geopolymers and favors mechanical strength [25]. As a result, the compressive strength of the SiCSMG samples increased with increasing NS/SS ratio (Figure 2). The compressive strength of the samples with an NS/SS ratio of 1.6 was because increasing the NS/SS ratio promoted the dissolution of the initial solid and the elevated reactivity. Yaghoubi et al. [26] noted that the geopolymer strength was related to the degree of polycondensation, which was strongly influenced by the soluble SiO_4_ and AlO_4_ content of the polymer system. A high degree of polycondensation in the Si–O–Al framework structure corresponds to high strength [27]. As shown in the FTIR analysis, an NS/SS ratio of 1.2–1.6 in the SiCSMGs with a SiCS replacement ratio of 10% leads to a peak from the Si–O–T bond (asymmetric type) stretching at 1037 cm^−1^ transfer to 1027 cm^−1^. It confirmed the development of polysialate and crosslinking network formation that overlaid a chain of polysilicate [28]. The results indicated that dissolution of the initial solid and the elevated reactivity increased with the NS/SS ratio of 1.6. Moreover, the compressive strength of the 10% SiCS replacement level was higher than that of all the other samples. At an NS/SS ratio of 1.6, the SiCSMG with 10% SiCS content had higher strength of about 70 MPa after 56 days curing. Hu et al. [13] noted that the synergistic effect was suggested as contributing to the strength of the geopolymer, which reached the maximum of 23.8 MPa at the curing age of 28 days when the FA content was 50 % [13]. The synergistic effect of the SiCS and MK promoted the reaction progression, which caused increasing amounts of amorphously structured geopolymeric gels in the geopolymerized system [13], which is consistent with the abovementioned research.

## 5. Conclusions

The principal chemical components in the SiCS were 51.35% Si, 6.90% C, and 0.42% Al; those in the MK were 24.21% Si and 22.77% Al. After curing for up to 28 days, SiCSMGs with 10%–20% SiCS content had bulk density 1.16–1.22 g/cm^3^. It was known that the bulk density increased with curing age and additional SiCS. The porosity of the 10% SiCSMG with an NS/SS ratio of 1.6 was 48.53%; this may be attributable to the geopolymer products filling the geopolymer pores. For an NS/SS ratio of 1.6, replacing 40% of the SiCSMG with SiCS resulted in an extension of setting time (initial besides final) to be 221 min and 285 min, one-to-one. Therefore, the high SiCS content of geopolymer sample may reduce the precursor amounts and delay the geopolymer reaction. At an NS/SS ratio of 1.6, the SiCSMG with 10% SiCS content had higher strength of about 70 MPa after 56 days curing. Because of the synergistic effect of the SiCS, MK promoted the reaction progression with favorable mechanical strength. At a high NS/SS ratio, the Si–O–T asymmetric type bond stretching at 1037 cm^−1^ (in the spectrum after 1 day of curing) was shifted to 1027 cm^−1^ after 28 days of curing because of the accelerated dissolution and participation of unreacted particles during geopolymerization. In summary, this study demonstrated that a geopolymer can be formed with a SiCS substitution of 10% and with an NS/SS ratio of 1.6, and that this geopolymer is a feasible material. The novelty of this paper is to provide the effect of an alkaline-activator solution on a geopolymer reaction of SiC sludge-based geopolymers (SiCSMG). For future work, we expect to analyze other important properties of SiC sludge-based geopolymers by heat evolution, differential thermal analysis (DTA/TG), and nuclear magnetic resonance (NMR).

## Figures and Tables

**Table 1:** An elementary analysis of materials.

Composition	SiCS	MK	Kaolinite
Si (%)	51.35 ± 0.01	24.21 ± 0.01	25.10 ± 0.01
Al (%)	0.42 ± 0.01	22.77 ± 0.01	20.06 ± 0.01
Fe (%)	0.41 ± 0.01	0.91 ± 0.01	0.62 ± 0.01
Ca (%)	0.06 ± 0.01	0.18 ± 0.01	0.14 ± 0.01
Mg (%)	N.D. ^1^	N.D. ^1^	N.D. ^1^
S (%)	0.02 ± 0.01	N.D. ^1^	N.D. ^1^
Na (%)	N.D. ^1^	0.03 ± 0.01	0.03 ± 0.01
K (%)	0.01 ± 0.01	0.27 ± 0.01	0.28 ± 0.01
C (%)	6.90 ± 0.01	N.D. ^1^	N.D. ^1^

^1^ N.D.: not detected; n = 3, mean ± standard deviation.

**Table 2:** Setting time of different NS/SS ratios with SiCSMGs.

NS/SS Ratio	Replacement (%)	Initial Setting (min)	Final Setting (min)
0.8	0	41	90
	10	74	105
	20	83	120
	30	96	135
	40	132	210
1.2	0	65	120
	10	89	150
	20	125	180
	30	128	180
	40	145	210
1.6	0	101	150
	10	134	180
	20	158	225
	30	192	255
	40	221	285

**Table 3:** FTIR spectra result of SiCSMGs.

Characteristics Vibrations	Wavenumber (cm^–1^)
Bending vibration (H–O–H)	1642
Stretching vibration (O–C–O)	1460
Asymmetric stretching (Si–O–T) (T = Al or Si)	900–1300
Stretching (T–OH) (T = Al or Si)	860
Bending (Al–O–Si)	700
Bending (Si–O–Si)	470
